# Leptin Receptor b (LEPRb) Mutations Disrupt Hypothalamic Control of the Reproductive Axis

**DOI:** 10.3390/ijms27052482

**Published:** 2026-03-08

**Authors:** Athanasios Zikopoulos, Efthalia Moustakli, Periklis Katopodis, Vasilis Sebastian Paraschos, Anastasios Potiris, Ismini Anagnostaki, Aikaterini Lydia Vogiatzoglou, Konstantinos Zacharis, Theodoros Karampitsakos, Konstantinos Zikopoulos, Sofoklis Stavros

**Affiliations:** 1 Department of Reproductive Medicine and Surgery, University College London Hospitals NHS Foundation Trust, 235 Euston Road, London NW1 2BU, UK; thanzik92@gmail.com; 2 Department of Nursing, School of Health Sciences, University of Ioannina, 4th Kilometer National Highway Str. Ioannina-Athens, 45500 Ioannina, Greece; ef.moustakli@uoi.gr; 3 Laboratory of Medical Genetics in Clinical Practice, Faculty of Medicine, School of Health Sciences, University of Ioannina, 45110 Ioannina, Greece; katopodisper@gmail.com; 4 Department of Obstetrics and Gynecology, Corewell Health Hospital, 4700 Schaefer Road Suite 310, Dearborn, MI 48126, USA; vasilispar10@gmail.com; 5 Third Department of Obstetrics and Gynecology, University General Hospital “ATTIKON”, Medical School, National and Kapodistrian University of Athens, 12462 Athens, Greece; apotiris@med.uoa.gr (A.P.); vogiatzoglou.lydia@gmail.com (A.L.V.); theokarampitsakos@hotmail.com (T.K.); sfstavrou@med.uoa.gr (S.S.); 6 Medical School, National and Kapodistrian University of Athens, 11528 Athens, Greece; isanagnostaki3@gmail.com; 7 Department of Obstetrics and Gynecology, General Hospital of Lamia, 35100 Lamia, Greece; zaxarisk@yahoo.com; 8 Department of Obstetrics & Gynecology, University Hospital of Ioannina, 45500 Ioannina, Greece

**Keywords:** JAK2/STAT3 signaling, central leptin resistance, Kiss1, gonadotropin-releasing hormone, hypothalamic circuitry, pubertal failure, infertility, metabolic–reproductive integration, receptor signaling defects, neuroendocrine regulation

## Abstract

Adipocytes produce the hormone leptin, a hormone that links energy availability to reproductive function by permitting activation of the hypothalamic–pituitary–gonadal (HPG) axis. Loss-of-function mutations in the long leptin receptor isoform (LEPRb) disrupt intracellular signaling pathways, including the Janus kinase 2 (JAK2)/signal transducer and activator of transcription 3 (STAT3), phosphoinositide 3-kinase (PI3K), and mitogen-activated protein kinase (MAPK) pathways, resulting in central leptin resistance and impaired neuroendocrine control of reproduction. Evidence from human monogenic obesity syndromes, animal models, and neuroendocrine studies indicates that LEPRb mutations disrupt hypothalamic circuitry upstream of gonadotropin-releasing hormone (GnRH) neurons, impairing GnRH pulsatility and leading to hypogonadotropic hypogonadism (HH) and infertility. This review synthesizes molecular, translational, and clinical data highlighting the central role of kisspeptin-mediated signaling in leptin-dependent reproductive regulation. Current therapeutic limitations are discussed alongside emerging approaches, including kisspeptin-based therapies and receptor-targeted strategies. Elucidating how LEPRb dysfunction disrupts metabolic–reproductive integration may provide insights into both rare monogenic conditions and common obesity-associated reproductive dysfunction.

## 1. Introduction

From an evolutionary perspective, reproductive function is tightly coupled to energy availability, and fertility is activated only when sufficient metabolic resources are available [1]. Over recent decades, leptin has been recognized as a key hormone that signals energy availability to the central nervous system. Produced predominantly by white adipose tissue, leptin permits activation and maintenance of the HPG axis, thereby coordinating energy balance and reproductive function [2,3].

Multiple isoforms of the class I cytokine receptor known as the LEPRb influence the biological actions of leptin. Hypothalamic areas implicated in energy expenditure, appetite regulation, and neuroendocrine control have significant levels of expression of the long signaling isoform, LEPRb. LEPRb is essential for intracellular signal transduction and, upon activation, engages multiple downstream signaling pathways, including JAK2–STAT3, PI3K, and MAPK signaling [4,5]. Collectively, these pathways coordinate neuronal activity and transcriptional regulation underlying metabolic homeostasis. Leptin indirectly controls reproductive function through intermediary neuronal populations such as kisspeptin-producing kisspeptin/neurokinin B/dynorphin (KNDy) neurons, as functional LEPRb is not produced in GnRH neurons [6].

Genetic abnormalities in the leptin–LEPRb signaling system shed light on its physiological role in reproductive development. The three most prevalent clinical signs of loss-of-function LEPR mutations are infertility, HH, and severe early-onset obesity [7,8]. Despite normal or high levels of circulating leptin, LEPRb mutations alter receptor-mediated signaling, leading to significant central leptin resistance, disruption of the HPG axis, and delayed or missing puberty. In contrast, leptin replacement therapy is a successful treatment for congenital leptin insufficiency [9,10].

Clarifying the therapeutic consequences of defective leptin signaling has become more crucial due to the rising prevalence of obesity and metabolic diseases worldwide [9,11]. In addition to clarifying the processes behind uncommon genetic disorders, studies on monogenic LEPRb deficiency offer a more comprehensive understanding of leptin resistance in common metabolic diseases. Through the integration of genetic, clinical, and translational evidence, this review demonstrates how LEPRb mutations affect fertility by disrupting the generation of reproductive hormones [12,13].

There is still a lack of a thorough synthesis of the molecular processes by which LEPRb mutations impair the control of reproductive hormones and the hypothalamus circuitry, despite growing awareness of the metabolic–reproductive interaction. The purpose of this review is to integrate current evidence from molecular studies, animal models, and clinical investigations to delineate the mechanistic pathways linking LEPRb dysfunction to impaired GnRH pulsatility and HH, and to evaluate emerging therapeutic strategies targeting downstream neuroendocrine signaling.

This review is structured into four major thematic sections. First, we examine the molecular architecture and intracellular signaling pathways of LEPRb. Second, we analyze hypothalamic neurocircuitry linking leptin sensing to GnRH pulsatility. Third, we synthesize clinical evidence from human monogenic LEPRb deficiency and animal models. Finally, we evaluate current and emerging therapeutic strategies, highlighting translational challenges and future research directions.

## 2. Literature Search Strategy

This narrative review synthesizes current evidence regarding the role of LEPRb signaling in the integration of metabolic status and reproductive function. Relevant literature was identified through structured searches of PubMed, Scopus, and Web of Science using combinations of keywords including “leptin receptor,” “LEPRb mutations,” “hypogonadotropic hypogonadism,” “monogenic obesity,” “kisspeptin,” “GnRH,” “KNDy neurons,” “leptin resistance,” “HPG axis,” “puberty,” and “reproductive dysfunction.”

Priority was given to peer-reviewed original research articles, translational investigations, clinical cohort studies, and mechanistic experimental studies published primarily between 2000 and 2025. Seminal earlier studies were included where necessary to provide foundational mechanistic context regarding leptin signaling pathways and hypothalamic regulation of reproduction.

Studies were selected based on their relevance to molecular mechanisms of LEPRb signaling, genotype–phenotype correlations, neuroendocrine regulation of GnRH pulsatility, and therapeutic strategies targeting downstream pathways. Additional publications were identified through manual screening of reference lists of selected articles.

The retrieved literature was synthesized thematically according to molecular mechanisms, hypothalamic circuitry, clinical manifestations, and therapeutic implications to support the integrative framework presented in this review. No new experimental data were generated or analyzed.

## 3. Physiology of Leptin–LEPRb Signaling in Reproductive Endocrinology

Leptin is an essential metabolic signal that connects the neuroendocrine systems that control fertility to the body’s energy reserves. Adipocytes create the majority of the circulating leptin, which indicates long-term energy availability. It rises with fat storage and falls during times of energy deficiency [14,15,16]. When energy stores are sufficient, leptin signaling facilitates HPG axis activation and supports normal reproductive function. Reduced leptin levels cause the release of reproductive hormones to be suppressed during periods of fasting, prolonged sickness, or malnutrition. Crucially, leptin functions as a permissive signal necessary for regular gonadotropin secretion and reproductive competence rather than directly initiating reproduction [15].

### 3.1. Leptin as an Energy Availability Signal

Leptin enters the bloodstream, crosses the blood–brain barrier, and binds to receptors in key hypothalamic nuclei to transmit information regarding long-term energy adequacy [17,18]. The normal GnRH pulsatility, which is necessary for the downstream release of FSH and LH, is maintained by adequate leptin signaling. Reduced leptin levels rapidly suppress GnRH pulsatility and gonadotropin secretion during caloric restriction or increased energy expenditure. This adaptive response suppresses reproductive activity when metabolic resources are insufficient [19,20,21].

### 3.2. LEPRb Structure and Intracellular Signaling Pathways

**Figure 1:**
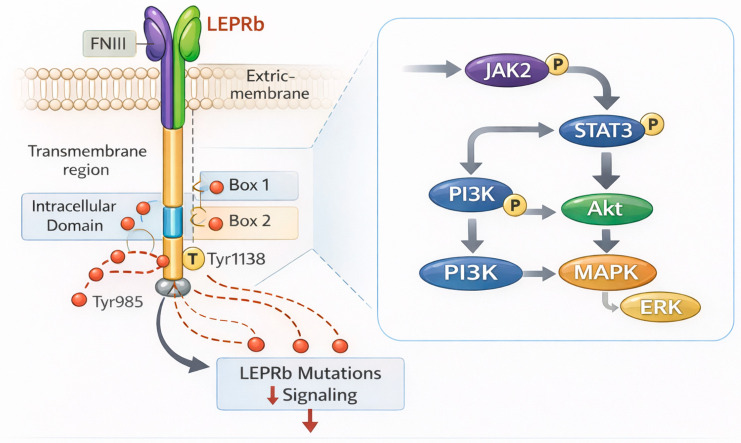
Structural and functional domains of LEPRb and key signaling nodes affected by pathogenic mutations. Schematic of the long signaling isoform of the LEPRb, showing the extracellular FNIII domain, transmembrane segment, and intracellular Box 1/Box 2 motifs with key tyrosine residues (e.g., Tyr1138, Tyr985). Leptin binding activates JAK2-mediated phosphorylation, leading to STAT3, PI3K/Akt, and MAPK/ERK signaling. Pathogenic mutations reduce phosphorylation-dependent signaling. Abbreviations: P, phosphorylation; T (Tyr), tyrosine residue.

Encoded by the LEPRb gene, the LEPRb is a member of the class I cytokine receptor family and is expressed in several tissues, with the hypothalamus being the primary site of its most important functions [22]. LEPR exists as multiple isoforms generated by alternative splicing, which can be broadly grouped into long, short, and soluble forms. The long signaling isoform, LEPRb, contains the full intracellular domain required for JAK2 recruitment and downstream activation of STAT3, PI3K/Akt, and MAPK/ERK pathways, and it mediates most of leptin’s central metabolic and neuroendocrine effects. In contrast, the short isoforms (LEPRa, LEPRc, LEPRd, and LEPRf) possess truncated intracellular tails with limited signaling capacity; these isoforms are expressed in peripheral tissues and at barrier interfaces and are thought to contribute to leptin transport, clearance, and local regulatory actions rather than canonical JAK2/STAT3 transcriptional signaling. A soluble isoform (LEPRe) lacks the transmembrane domain and circulates as a leptin-binding protein that can modulate leptin bioavailability and kinetics. Although this review focuses primarily on reproductive consequences driven by impaired LEPRb signaling in the hypothalamus, isoform diversity is relevant for interpreting leptin distribution, receptor availability, and phenotype variability [23]. Leptin’s metabolic and neuroendocrine effects are mediated by its long signaling isoform, LEPRb, one of several receptor variants. The structural components of LEPRb include an extracellular leptin-binding domain, a single transmembrane segment, and an intracellular domain containing conserved Box 1 and Box 2 motifs required for signal transduction (Figure 1). These motifs facilitate recruitment and activation of JAK2 [3,24].

Leptin binding induces conformational changes in LEPRb that activate JAK2, leading to phosphorylation of downstream signaling molecules. Upon phosphorylation, STAT3 translocates to the nucleus to regulate transcription of genes involved in endocrine regulation and appetite control [24,25,26]. Leptin concurrently activates the PI3K–Akt pathway, which regulates neuronal excitability and synaptic transmission, as well as the MAPK/ERK pathway, which promotes neuronal survival, plasticity, and feedback regulation. Together, these signaling cascades allow the hypothalamus to incorporate metabolic data and control neuroendocrine output. Defects in receptor binding, intracellular signaling, transcriptional regulation, or other components of this signaling cascade can impair leptin-mediated reproductive control [27,28,29]. Table 1 provides a summary of the primary structural domains of LEPRb and the signaling roles they play.

### 3.3. Hypothalamic Integration and Indirect Modulation of GnRH Neurons

Since kisspeptin signaling is the primary downstream mediator that connects leptin sensing to GnRH neuronal activation, this review discusses its physiological function, pathological disruption in LEPRb deficiency, and therapeutic implications across several sections.

**Figure 2:**
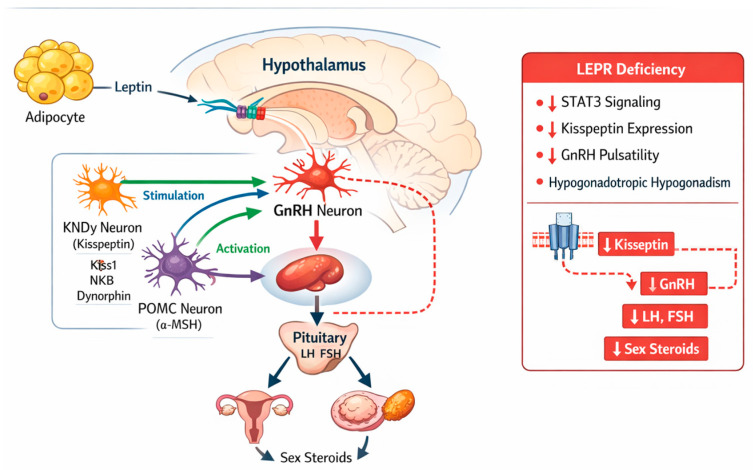
Schematic representation of leptin signaling in hypothalamic neuronal populations regulating the HPG axis. Circulating leptin binds to LEPRb expressed in KNDy and POMC neurons, promoting kisspeptin release and stimulation of GnRH neurons, leading to LH and FSH secretion and downstream gonadal steroid production. In LEPRb deficiency, impaired intracellular signaling reduces kisspeptin expression and GnRH pulsatility, resulting in HH. Solid arrows indicate stimulatory pathways, whereas dashed lines represent disrupted signaling in LEPRb deficiency. *Abbreviations:* HPG, hypothalamic–pituitary–gonadal; LEPRb, long isoform of the leptin receptor; KNDy, kisspeptin/neurokinin B/dynorphin; POMC, proopiomelanocortin; GnRH, gonadotropin-releasing hormone; LH, luteinizing hormone; FSH, follicle-stimulating hormone.

The absence of functional LEPRb expression in GnRH neurons is a defining feature of leptin’s reproductive effects, indicating that leptin indirectly regulates reproductive function. Instead, the GnRH network receives metabolic information from upstream hypothalamic neuronal populations influenced by leptin [21]. Among the most important are KNDy neurons, which generate the pulsatile release of GnRH and co-express dynorphin, kisspeptin, and neurokinin B. The onset of puberty and maintenance of adult reproductive function depend on leptin-dependent stimulation of kisspeptin production within this network [35,36]. The central integration of these hypothalamic circuits is illustrated in Figure 2.

Leptin increases Kiss1 gene expression, stimulates kisspeptin release, and stabilizes GnRH pulse frequency and amplitude through LEPRb-mediated signaling in these intermediary neurons. Normal gonadal steroidogenesis and gametogenesis are thereby enabled through appropriate LH and FSH secretion from the anterior pituitary. This signaling cascade supports spermatogenesis and testosterone synthesis in males and is essential for follicular growth and ovulatory function in females. Leptin coordinates metabolic and reproductive signaling, ensuring activation of the reproductive axis only under conditions of sufficient energy availability [15,20]. Although substantial evidence supports the role of KNDy neurons as intermediaries linking leptin signaling to GnRH pulsatility, some experimental studies suggest that leptin may also influence reproductive function through additional hypothalamic nodes, including the ventral premammillary nucleus. These findings indicate that the precise neuronal pathways mediating leptin’s reproductive effects remain incompletely defined and may involve parallel circuits.

## 4. LEPR Mutations: Molecular Mechanisms Impacting Reproduction

**Figure 3:**
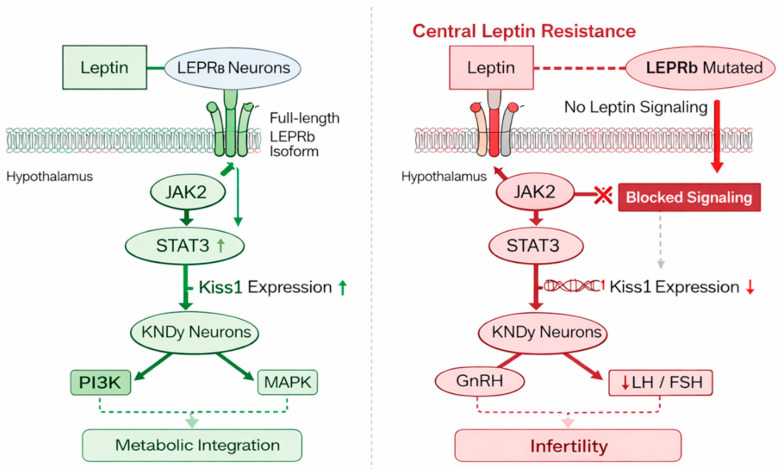
Normal LEPRb signaling versus LEPRb deficiency in the hypothalamic control of reproduction. Under physiological conditions (**left**), leptin activates hypothalamic LEPRb, stimulating JAK2/STAT3 signaling, increasing Kiss1 expression in KNDy neurons, and promoting GnRH pulsatility and LH/FSH secretion. In LEPRb deficiency (**right**), receptor dysfunction impairs downstream signaling, reduces Kiss1 expression, suppresses GnRH release, and results in HH.

The integration of metabolic and reproductive function can be disrupted by single-gene abnormalities, and mutations in the LEPRb gene represent one of the clearest examples. Despite elevated circulating leptin levels, individuals with LEPRb deficiency typically present with severe early-onset obesity, hyperphagia, significant HH, and markedly reduced reproductive hormone levels [7,37]. This phenotype reflects near-complete central leptin resistance, in which the hypothalamus fails to respond adequately to leptin signaling (Figure 3). Reproductive dysfunction arises from defects at multiple levels, including impaired intracellular signaling, structural alterations of the receptor, disrupted hypothalamic neuronal communication, and failure of downstream hormonal regulation [38].

### 4.1. Types and Functional Classes of LEPRb Mutations

More than 80 pathogenic or likely pathogenic variants of the LEPRb gene have been identified to date. The extracellular leptin-binding domain or intracellular regions necessary for interaction with JAK2 are frequently affected by missense mutations, which alter individual amino acids in critical areas of the receptor [39,40]. Frameshift and nonsense mutations disrupt the reading frame or introduce premature stop codons, resulting in truncated, nonfunctional receptor proteins. Larger deletions or structural rearrangements may remove essential domains or completely abolish expression of the long signaling isoform, LEPRb, whereas splice-site mutations disrupt normal RNA processing and impair proper receptor assembly [39,41,42].

Leptin-mediated neuroendocrine control depends on LEPRb, which is affected by the majority of disease-causing mutations. Pathogenic LEPRb variants often cluster in the intracellular Box 1 and Box 2 motifs required for JAK2 recruitment and downstream signaling, as well as in the fibronectin type III (FNIII) domains critical for leptin binding. The major functional classes of LEPRb mutations and their molecular and reproductive consequences are summarized in Table 2 [10,43,44].

### 4.2. Consequences for Receptor Structure, Trafficking, and Signaling

Leptin signaling is disrupted by pathogenic LEPRb mutations through multiple molecular mechanisms. Some variants impair proper receptor folding or post-translational processing, leading to endoplasmic reticulum retention and defective cell-surface trafficking. Even when circulating leptin levels are normal or elevated, leptin cannot bind efficiently to its receptor. However, additional mutations directly change the extracellular leptin-binding domain, lowering receptor affinity for leptin, even when membrane localization is successful [49,50].

Further mutations impact LEPRb’s intracellular domain, reducing its capacity to engage with JAK2 or start subsequent phosphorylation processes. Consequently, MAPK signaling is compromised, the PI3K–Akt pathway is not activated, and STAT3 fails to translocate to the nucleus to regulate leptin-responsive gene expression [17,27,41,51]. On the other hand, the relative contribution of individual signaling pathways (JAK2/STAT3 vs. PI3K/Akt or MAPK/ERK) to reproductive dysfunction remains debated. While STAT3 signaling has been strongly linked to neuroendocrine regulation of reproduction, experimental models suggest that parallel signaling routes may partially compensate under certain conditions, highlighting the complexity of leptin receptor signaling networks. Collectively, these abnormalities markedly reduce leptin-dependent transcriptional activity in hypothalamic neurons.

Despite receptor malfunction, adipocytes continue to manufacture leptin proportionate to body fat growth, which raises the amount of leptin in the blood. However, the brain continues to perceive a state of energy deficiency due to ineffective central leptin signaling. This mismatch exacerbates metabolic dysfunction, promotes progressive obesity, and drives chronic hyperphagia, thereby reinforcing a cycle of leptin resistance [52,53].

### 4.3. Effects on Hypothalamic Circuits Regulating the HPG Axis

The disturbance of hypothalamic neural networks upstream of GnRH neurons is the main cause of reproductive failure in LEPRb deficiency [54]. Among the most impacted are KNDy neurons that produce kisspeptin, which typically integrate metabolic information to regulate GnRH pulsatility. Under normal circumstances, leptin increases the expression of the Kiss1 gene and the release of kisspeptin, which stimulates rhythmic GnRH pulsatility. The disruption of GnRH pulsatility, which arises from decreased kisspeptin production, decreased KNDy neuronal responsiveness, and insufficient stimulatory input to GnRH neurons in the absence of functioning LEPRb signaling, is a characteristic of HH [55,56,57].

Furthermore, proopiomelanocortin (POMC) neurons and neuropeptide Y/agouti-related peptide (NPY/AgRP) are important for maintaining energy homeostasis, which in turn indirectly controls reproductive function [58,59]. However, the development of leptin resistance affects these neurons. Reproductive health benefits from lower POMC neuronal activity and α-MSH synthesis, which are caused by LEPRb mutations. GnRH receives more inhibitory inputs when NPY and AgRP neuronal function is elevated. This interferes with the control of the release of reproductive hormones [60,61,62].

### 4.4. Downstream Disruption of Gonadotropin and Sex Steroid Secretion

The pituitary gland and the gonads are both impacted by a cascade that is started when leptin signaling fails at the hypothalamus level. Reduced LH and FSH production as a result of decreased GnRH stimulation causes HH, which is characterized by low or abnormally normal gonadotropin levels. Males produce less testosterone as a result of poor gonadal steroidogenesis, whereas females produce less progesterone and estradiol [63,64,65].

This endocrine disorder manifests as infertility, amenorrhea, persistent anovulation, and delayed follicular development in females. In males, the consequences of inadequate gonadotropin and androgen signaling include cryptorchidism, low levels of circulating testosterone, delayed or incomplete pubertal development, and impaired spermatogenesis. These findings emphasize the essential role of intact LEPRb signaling in reproductive hormone production and fertility [15,66,67].

## 5. Clinical Evidence Linking LEPRb Mutations to Reproductive Dysfunction

Clinical findings in people with mutations in the LEPRb gene show how crucial leptin receptor signaling is to human reproductive physiology. Patients with biallelic loss-of-function LEPRb mutations typically present clinically with severe early-onset obesity, chronic hyperphagia, and significant HH. The hypothalamus is essentially in a state of functional energy deprivation in these people because they lack efficient central leptin signaling despite having normal or noticeably raised circulating leptin levels. The crucial function of LEPRb-mediated signaling in the reproductive axis is highlighted by the consistent reproductive failure observed across age groups, sexes, and mutation types [44,45].

### 5.1. Phenotypic Presentation in Human Monogenic LEPRb Deficiency

Early infancy is when monogenic LEPRb deficiency first appears as a unique constellation of clinical symptoms. Severe obesity, typically present within the first year of life, is characterized by persistent hyperphagia and reduced satiety due to impaired central leptin sensing. The HPG axis is dysfunctional during adolescence when pubertal growth is absent or delayed. HH is frequently identified on endocrine evaluation, with LH and FSH levels insufficient to sustain normal sexual maturation. Infertility or markedly reduced fertility often persists into adulthood [68,69].

Hormonal profiles typically show decreased circulating sex steroid levels, such as testosterone in males and estradiol in females, together with low or inappropriately normal gonadotropin concentrations. Importantly, normal hormonal responses to exogenous GnRH stimulation indicate preserved pituitary structure and intrinsic responsiveness. LEPR deficiency is distinguished from congenital leptin deficiency by a marked dissociation between elevated circulating leptin levels and lack of physiological responsiveness, despite the efficacy of leptin replacement therapy in the latter condition [70,71,72].

### 5.2. Sex-Specific Reproductive Manifestations

Although disruption of the HPG axis is a shared feature, males and females with LEPR mutations exhibit distinct clinical manifestations. Reproductive development is often severely impaired in females [12]. In addition to failure to develop typical secondary sexual characteristics, such as breast development, most affected individuals present with primary amenorrhea. This leads to chronic anovulation because of impaired ovarian maturation. Fertility seldom occurs on its own without medical assistance, and hormonal tests usually show low levels of progesterone and estradiol. Reproductive function does not resume unless hormone replacement techniques are used [73,74,75].

Due to inadequate fetal testosterone production, males with LEPRb deficiency often show early indicators of decreased androgen exposure, such as micropenis and cryptorchidism. Affected males usually have decreased testicular volume, inadequate development of secondary sexual traits, and delayed or missing pubertal growth during adolescence [76]. Endocrine evaluation reveals low testosterone levels, decreased LH and FSH secretion, and often markedly impaired spermatogenesis. Fertility is commonly diminished among these patient groups, although there is partial responsiveness to pulsatile GnRH or hormone supplementation therapy [77,78].

### 5.3. Cohort Studies and Genotype-Phenotype Correlations

Longitudinal cohort studies provide important insight into the reproductive consequences of LEPRb mutations. Children with homozygous or compound heterozygous LEPR mutations do not undergo spontaneous puberty and require medical intervention to induce sexual maturation. Similarly, adults with untreated LEPRb impairment continue to show HH even after extended exposure to severe obesity, indicating that increased energy storage alone cannot compensate for reduced leptin receptor signaling [12,79]. For instance, it has been established that the correlation between genotypes and phenotypes reveals that the “extent of disruption of the signaling event caused by particular mutations correlates inversely with the severity of reproductive dysfunction. Disruption of critical signaling events involving the recruitment of JAK2 or activation of STAT3 is associated with more severe endocrine abnormalities [80,81]. Reported clinical cohorts demonstrate that the severity of reproductive dysfunction correlates with the degree of disruption of intracellular LEPRb signaling. Nevertheless, current genotype–phenotype correlations remain incomplete. Functional characterization of many LEPR variants is still lacking, and discrepancies between predicted molecular defects and clinical presentation suggest that additional genetic modifiers or environmental factors may influence reproductive outcomes. Representative genotype–phenotype associations are summarized in Table 3.

Across reported cohorts, reproductive phenotypes show substantial variability even among individuals with biallelic LEPRb variants, suggesting incomplete penetrance for some traits and a role for modifying factors such as background genetics, degree of residual receptor expression/trafficking, and cardiometabolic comorbidities. This heterogeneity underscores the need for standardized phenotyping, longitudinal reproductive follow-up, and systematic aggregation of variant-level functional data to strengthen genotype–phenotype prediction [45].

Notably, clinical variability persists even among individuals carrying similar or identical LEPR mutations, suggesting the contribution of modifier genes, environmental influences, and differences in residual receptor signaling. The degree of STAT3 pathway disruption may partially predict severity; however, systematic functional classification of variants remains incomplete. Expanded genotype-driven phenotyping studies are needed to refine predictive models and improve counseling strategies [10,44,71].

### 5.4. Evidence from Animal Models Supporting the Human Phenotype

The clinical observations in humans with LEPRb deficiency are well supported by findings from animal models [2,82]. The db/db model and other LEPRb-deficient mice have been shown to share the same phenotypes with the human population. Mice with this mutation have lower gonadotropin levels, are infertile, have delayed sexual maturation, and have elevated levels of leptin. These animal models demonstrate that leptin availability alone is insufficient to maintain reproductive function [67,83,84].

Kisspeptin injection can partially restore luteinizing hormone output, according to experimental experiments conducted in animals lacking LEPRb, which further supports the idea that compromised kisspeptin signaling is a major cause of reproductive failure [35,85]. Furthermore, it is confirmed by neuron-specific deletion studies that leptin conducts its reproductive effects through upstream hypothalamic circuits rather than directly acting on GnRH neurons [54,86]. Collectively, these studies substantiate the molecular relationships among hypothalamic integration, LEPRb signaling, and reproductive hormone regulation in human disease.

## 6. Downstream Effects on the Reproductive Axis

However, when the signaling pathway is disrupted, a series of disorders that impact the HPG axis are initiated, impairing several crucial elements of reproductive development and function. The hypothalamus and the gonads are among these abnormalities since the system is unable to properly coordinate or regulate the secretion of metabolic and reproductive hormones [87,88,89]. Therefore, both males and females with LEPRb deficiency have complete abnormalities including gonadotropin production, sexual steroids, and pubertal onset.

### 6.1. Failure of Pubertal Initiation and Progression

Leptin acts as a permissive signal for pubertal development, facilitating the developmental increase in GnRH pulsatility, a process that is largely driven by enhanced kisspeptin signaling [15]. The mechanism underlying LEPRb mutations is fundamentally defective. Impaired expression of the Kiss1 gene limits kisspeptin availability, preventing adequate stimulation of GnRH release. As a result, GnRH pulsatility fails to increase during childhood and adolescence, and LH and FSH secretion remains suppressed [90].

The absence of this coordinated neuroendocrine activation prevents normal gonadal maturation, leading to absent or markedly delayed puberty, lack of secondary sexual characteristic development, and persistence of prepubertal hormonal profiles. Spontaneous pubertal development does not occur without medical intervention in most patients [91,92].

### 6.2. HH

A more widespread and long-lasting malfunction in gonadotropin secretion is reflected in the failure of pubertal activation in LEPRb deficiency [93]. Chronically decreased LH and FSH secretion results from impaired leptin signaling, which weakens the hypothalamic drive necessary to maintain pulsatile GnRH release after puberty [94]. Despite significant reproductive damage, this endocrine condition presents as HH, which is defined by low or abnormally normal gonadotropin levels [95].

Throughout adolescence and adulthood, inadequate secretion of reproductive hormones persists in the absence of treatment. Hormonal therapy is consequently necessary to sustain reproductive endocrine function over time as well as to start pubertal development [69,96].

### 6.3. Ovarian Dysfunction in LEPRb Deficiency

In females, ovarian physiology is significantly impacted when leptin–LEPRb signaling is disrupted [97]. Chronic anovulation results from halted follicular development caused by insufficient LH and FSH secretion, which keeps follicles from maturing to the preovulatory stage. Estradiol and progesterone levels in the blood are consistently low as a result of poor ovarian steroidogenesis [98].

Infertility, primary amenorrhea, and lack of menstrual cyclicity are clinical manifestations of these abnormalities. Insufficient gonadotropin stimulation results in prepubertal functional activity even though ovarian morphology may appear anatomically normal. Usually, hormone replacement treatment or assisted reproductive technologies are required to restore reproductive function [99,100].

### 6.4. Testicular Dysfunction in Males

Through insufficient gonadotropin stimulation, male testicular development and endocrine function are disrupted by poor leptin receptor signaling [101]. Incomplete or delayed pubertal development and decreased testicular volume are the results of impaired Leydig cell testosterone synthesis caused by decreased LH signaling. Spermatogenesis is sometimes substantially impaired, and many affected individuals have pronounced oligospermia or azoospermia [102,103].

Micropenis and cryptorchidism are examples of congenital symptoms that may be exacerbated by inadequate androgen exposure during early life, particularly the newborn “minipuberty” period [104]. Although exogenous gonadotropins or pulsatile GnRH can sometimes partially restore fertility, the effectiveness of treatment varies and is frequently restricted in patients with total LEPRb impairment [21].

### 6.5. Broader Neuroendocrine Consequences

Given the crucial role leptin plays in regulating the metabolic and neuroendocrine systems, the effects of LEPRb mutations go beyond the reproductive axis. Disrupted hypothalamic signaling may alter thyroid hormone regulation, potentially affecting basal metabolic rate and energy expenditure [9,105]. Growth hormone secretion may also be dysregulated, further underscoring impaired integration between energy availability and the endocrine regulation of growth and development [106].

Furthermore, metabolic issues like insulin resistance and dyslipidemia are frequently seen in people with LEPRb deficiency. These disturbances not only increase cardiometabolic risk but may also exacerbate reproductive dysfunction and complicate hormonal treatment strategies [107,108]. Collectively, these findings underscore the systemic effects of leptin receptor dysfunction and the need for a comprehensive, interdisciplinary therapeutic approach.

## 7. Therapeutic Approaches

Therapeutic strategies targeting LEPRb-associated reproductive dysfunction can be categorized into (i) hormone replacement approaches that bypass central leptin signaling, (ii) downstream neuroendocrine stimulation strategies such as kisspeptin analogs, and (iii) emerging receptor-targeted or gene-based interventions currently under preclinical investigation.

Management of reproductive dysfunction associated with LEPRb mutations is challenging because the primary defect lies in the receptor itself rather than in circulating leptin levels. In contrast, leptin replacement therapy restores fertility, pubertal development, and appetite regulation in individuals with congenital leptin deficiency. Circulating leptin levels in LEPRb deficiency are typically normal or elevated, whereas intracellular signaling remains impaired, rendering leptin biologically ineffective [22].

Therefore, therapeutic strategies must either ignore the faulty receptor or actively stimulate downstream, intact reproductive axis components. To compensate for impaired hypothalamic signaling, current and emerging strategies focus on stimulating gonadotropin or GnRH pathways or targeting alternative neuroendocrine mechanisms [19,88].

### 7.1. Leptin Replacement Therapy and Its Limitations

People with congenital leptin deficit, in which the hormone itself is lacking, have shown great success with recombinant leptin therapy, particularly metreleptin [109]. In these situations, leptin replacement helps to restore the release of reproductive hormones, normalize appetite, and encourage pubertal growth. This treatment strategy, however, does not work in LEPRb deficiency, when the underlying issue is not leptin insufficiency but rather defective receptor-mediated signal transduction [9].

Hypothalamic signaling pathways are not activated by even supraphysiological levels of exogenous leptin due to the impairment of LEPRb activity [11]. Clinical data consistently show that in people with LEPRb mutations, leptin treatment does not increase gonadotropin secretion, pubertal development, or hunger management. Recombinant leptin (metreleptin) is an established therapy for congenital leptin deficiency; however, it has no demonstrated efficacy in individuals with biallelic LEPRb mutations due to receptor-level signaling failure. The aforementioned observations highlight the need for therapeutic approaches that function beyond leptin-LEPRb binding [110].

### 7.2. Leptin Receptor Agonists and Emerging Molecular Therapies

The limitations of conventional leptin therapy have led to research into novel molecular strategies for restoring or preventing leptin receptor activation. Developing synthetic LEPRb agonists or customized leptin analogues that interact with certain receptor domains to enhance receptor activation is one tactic. Despite their conceptual potential, these drugs are still in the preclinical stage and have not yet been tried in patients with confirmed LEPRb deficiency [22,111].

In addition to leptin analogues, receptor-targeted biologics have recently emerged as a promising approach to restore LEPRb pathway activation. A fully human leptin receptor agonist monoclonal antibody (REGN4461; mibavademab) has demonstrated leptin-independent activation of human LEPRb signaling, with favorable metabolic effects in preclinical models and early clinical evaluation in leptin-pathway disorders. These data provide proof-of-concept that pharmacologic LEPRb activation may be achievable even when endogenous leptin signaling is ineffective, although the applicability to specific LEPRb loss-of-function variants will depend on mutation location and residual receptor expression [111].

Another potential treatment strategy is gene-based therapies. Although adeno-associated viral vector–mediated delivery of functional LEPRb to specific hypothalamic regions has demonstrated proof of concept in animal models, clinical translation remains constrained by major ethical and technological challenges [112,113,114]. In parallel, chemogenetic and optogenetic methods that do not require functional LEPRb have been used to selectively activate leptin-responsive hypothalamic circuits in experimental mice. Although these approaches are not yet translatable to clinical practice, they provide important insights into future therapeutic targets [5].

Gene-based approaches, including viral vector–mediated LEPRb delivery, remain at the preclinical stage. Although proof-of-concept studies demonstrate restoration of leptin signaling in animal models, translation to human therapy faces substantial barriers, including blood–brain barrier targeting, long-term safety monitoring, immunogenicity, and ethical considerations related to CNS gene modification.

### 7.3. Kisspeptin-Based Therapies

As a crucial downstream mediator of leptin’s reproductive effects, kisspeptin has become a particularly appealing therapeutic target in LEPRb deficiency. KNDy neurons that produce kisspeptin are an important regulatory node in the reproductive axis, acting as a primary regulator of GnRH pulsatility. Crucially, with LEPRb loss, GnRH neurons maintain their structural integrity despite the disruption of leptin signaling upstream, so kisspeptin-induced downstream activation makes sense [115,116,117].

Kisspeptin analogues have been shown in clinical trials to partially restore reproductive hormone pulsatility and boost LH secretion in associated disorders with decreased hypothalamic drive, such as functional hypothalamic amenorrhea [118]. Although data in individuals with genetically confirmed LEPRb mutations are limited, animal models of leptin resistance demonstrate that kisspeptin treatment can enhance gonadotropin secretion. Collectively, these findings, pending confirmation of long-term safety and efficacy in humans, suggest that kisspeptin-based therapies represent the most physiologically targeted strategy currently under investigation for LEPRb-associated reproductive dysfunction [116,119,120]. Kisspeptin analogs have progressed to early-phase clinical evaluation in disorders characterized by reduced hypothalamic GnRH drive, such as functional hypothalamic amenorrhea. However, their use in genetically confirmed LEPRb deficiency remains limited to theoretical rationale and animal model data. Key challenges include maintaining physiologic pulsatility, avoiding receptor desensitization, and determining optimal long-term dosing strategies [121].

### 7.4. Hormonal Replacement and Assisted Reproductive Strategies

Direct hormonal therapy, which completely avoids the faulty leptin signaling, is currently the most effective method of restoring reproductive function in patients with LEPRb mutations [37]. Usually, low doses of estradiol are used to induce puberty in females, which is then gradually increased to create menstrual cycles. Following pubertal induction, combined estrogen and progesterone therapy maintains normal hormonal function. Exogenous gonadotropins or pulsatile GnRH treatment can be utilized to promote follicular growth and ovulation if fertility is sought [92,122].

Testosterone replacement therapy is used to promote the development of secondary sexual traits in males and to improve overall pubertal development. Human chorionic gonadotropin (hCG) and recombinant FSH are often required to stimulate sperm production in those attempting to conceive. However, success rates differ, especially in situations of severe receptor malfunction, and complete fertility restoration is not assured [123,124,125].

### 7.5. Metabolic Intervention and Weight Management

Optimizing metabolic health may confer additional benefits, even though current metabolic therapies cannot correct the underlying leptin receptor deficiency. Interventions that focus on insulin sensitivity, weight stabilization, and systemic inflammation may improve endocrine function and increase sensitivity to hormonal therapies. Bariatric surgery has been considered in some cases for individuals with severe obesity; there is currently no evidence to support its impact on reproductive outcomes in LEPRb deficiency [126,127]. Consequently, metabolic interventions ought to be considered supporting elements of an all-encompassing, customized management strategy rather than curative treatments.

Despite promising mechanistic advances, translation of novel therapies for LEPRb deficiency remains challenging. Central nervous system delivery, long-term safety, and cost considerations represent major obstacles for gene-based or neuromodulatory interventions. Furthermore, the rarity of monogenic LEPRb deficiency limits large-scale clinical trials, complicating evidence generation. In the near term, hormone replacement and assisted reproductive strategies remain the most feasible clinical options, whereas receptor-targeted biologics and gene-editing approaches are likely to require extended preclinical validation and phased clinical development before widespread application [112,114,128].

## 8. Integrative Perspective and Future Directions

### 8.1. Central Leptin Sensing as a Determinant of Reproductive Competence

Leptin receptor mutations provide a unique biological model through which the integration of metabolic status and reproductive function can be examined. The evidence synthesized in this review highlights the central role of leptin-dependent activation of hypothalamic circuits in regulating gonadotropin output, GnRH pulsatility, and fertility [12]. LEPRb deficiency represents an extreme state of central leptin resistance and therefore offers a powerful framework for understanding how energy sufficiency is translated into reproductive competence [21].

One of the most striking findings emerging from studies of LEPRb dysfunction is the dissociation between circulating leptin levels and physiological response. Despite markedly elevated serum leptin concentrations, the hypothalamus behaves as though it is in a state of energy deprivation. These observations demonstrate that reproductive capability is determined not by absolute leptin levels but by effective central leptin sensing. When LEPRb signaling is impaired, kisspeptin expression declines, GnRH pulsatility becomes disrupted, and downstream gonadotropin secretion is suppressed [129,130].

The phenotype associated with LEPRb deficiency further underscores leptin’s indirect role in reproductive physiology. Functional LEPRb is absent from GnRH neurons; instead, leptin acts through intermediary neuronal populations, particularly KNDy neurons that co-express kisspeptin, neurokinin B, and dynorphin. Disruption of this finely regulated network destabilizes the entire HPG axis. Converging evidence from cellular experiments, animal models, and human clinical studies consistently supports this mechanistic paradigm [131,132].

### 8.2. Congenital LEPR Deficiency and Acquired Leptin Resistance

The clinical implications of LEPRb mutations extend beyond rare monogenic disorders. Although congenital LEPRb deficiency and obesity-associated leptin resistance differ mechanistically, they share important functional similarities. Both conditions are characterized by impaired hypothalamic leptin signaling, reduced GnRH drive, hypogonadism, and compromised fertility [133,134].

Congenital LEPRb mutations represent a genetically defined model of complete receptor dysfunction, whereas acquired leptin resistance in obesity likely involves post-receptor signaling defects, inflammation, endoplasmic reticulum stress, and altered neuronal responsiveness. Nevertheless, the reproductive phenotype observed in both settings suggests that intact leptin signaling is indispensable for maintaining HPG axis activity [7].

Understanding congenital LEPRb deficiency therefore provides mechanistic insight into broader metabolic–reproductive interactions. It highlights how chronic metabolic stress may compromise fertility in common clinical conditions, including obesity and metabolic syndrome, even in the presence of abundant energy stores [135].

Emerging evidence from recent clinical studies further underscores the complex interplay between metabolic dysfunction and reproductive outcomes. In women with polycystic ovary syndrome (PCOS), excessive body weight and insulin resistance independently and synergistically impair fertilization efficiency, embryo quality, and assisted reproductive outcomes. These findings highlight that metabolic–endocrine interactions extend beyond monogenic LEPRb deficiency and reinforce the broader concept that reproductive competence depends on integrated metabolic signaling. Incorporating metabolic profiling alongside hormonal assessment may therefore improve understanding and management of fertility impairments in both rare genetic and common metabolic conditions [136,137,138].

### 8.3. Therapeutic Implications and Future Research Directions

Despite significant advances in delineating the molecular mechanisms of LEPRb dysfunction, therapeutic success remains limited. Leptin replacement therapy, highly effective in congenital leptin deficiency, does not restore reproductive function in LEPRb mutations because the defect lies in receptor-mediated signal transduction. This distinction emphasizes the need for strategies that bypass or act downstream of the defective receptor [9,12,139]. Kisspeptin-based therapies represent a particularly promising approach, as they directly stimulate GnRH neurons while preserving physiological pulsatility. Although animal studies demonstrate partial restoration of gonadotropin secretion, robust clinical data in genetically confirmed LEPRb deficiency are still lacking. Long-term safety, sustained efficacy, and potential desensitization effects remain important unanswered questions [121].

Hormone replacement therapy currently remains the clinical cornerstone for inducing puberty and supporting fertility through exogenous gonadotropin administration [140]. However, these interventions do not correct the underlying neuroendocrine defect. Future strategies may involve receptor-targeted molecular therapies, gene-based interventions, or neuromodulatory approaches aimed at restoring hypothalamic circuit integrity [141].

More broadly, LEPRb mutations reveal fundamental constraints of the neuroendocrine system that integrates metabolism and reproduction [12]. Continued advances in molecular endocrinology, neurobiology, and gene therapy may enable the development of more targeted and mechanistically grounded treatments, not only for rare monogenic LEPR deficiency but also for reproductive dysfunction associated with common metabolic disorders [128,142].

Recent neurogenetic studies using modern circuit tools further refine leptin-responsive hypothalamic architecture and provide a translational framework for targeted interventions. Notably, CRISPR–Cas9 strategies have been used experimentally to disrupt Lepr in defined neuronal populations via viral delivery, enabling cell-type-specific dissection of leptin signaling and downstream phenotypes. In parallel, emerging chemogenetic and circuit-level modulation approaches continue to clarify how discrete leptin-responsive nodes influence neuroendocrine and reproductive outputs, supporting the longer-term feasibility of circuit-informed therapies [143,144].

### 8.4. Current Limitations and Knowledge Gap

Despite growing mechanistic and clinical insight into LEPRb-associated reproductive dysfunction, several important limitations remain. Most published reports involve small cohorts or single-family case studies, limiting the ability to establish robust genotype–phenotype correlations. Significant heterogeneity in reproductive presentation has been observed, even among individuals carrying similar variants, suggesting incomplete penetrance and the influence of genetic, epigenetic, or metabolic modifiers [145].

Longitudinal reproductive outcomes into adulthood remain insufficiently characterized, particularly with regard to fertility potential and response to hormonal or assisted reproductive interventions. Furthermore, standardized diagnostic criteria and management algorithms for LEPRb-related HH are lacking, resulting in variability in clinical evaluation and therapeutic strategies [146].

Future research should prioritize multicenter registries, systematic functional characterization of LEPRb variants, and long-term follow-up studies to better define mutation-specific reproductive trajectories and optimize individualized therapeutic approaches [44].

### 8.5. Clinical and Ethical Considerations

Advances in molecular and gene-targeted therapies for LEPRb deficiency raise important clinical and ethical considerations. Gene-based interventions and neuromodulatory approaches targeting central nervous system pathways require careful evaluation of long-term safety, potential off-target effects, and informed consent, particularly when proposed for pediatric patients. Ethical concerns related to germline modification, irreversible genomic changes, and equitable access to advanced therapies must be carefully addressed prior to clinical implementation [147].

Early genetic screening for LEPRb mutations may facilitate timely diagnosis, targeted hormonal management, and appropriate counseling for affected families. However, genetic testing also carries psychosocial implications, including stigmatization, uncertainty regarding prognosis, and challenges in variant interpretation. Structured genetic counseling is therefore essential [45].

Finally, accessibility and cost considerations represent significant barriers to the implementation of advanced biologics or gene-based therapies, particularly in rare monogenic disorders. Ensuring equitable access and developing standardized management frameworks will be critical as precision medicine approaches continue to evolve [148].

## 9. Conclusions

Reproductive function and energy availability are thus linked by the crucial pathway of leptin receptor signaling. Despite high levels of circulating leptin, loss-of-function mutations in the LEPRb gene cause substantial central leptin resistance, thereby impairing the central nervous system’s ability to recognize energy sufficiency. This deficiency causes HH, infertility, and a sustained decrease in gonadotropin output by impairing kisspeptin-mediated GnRH pulsatility.

The relevance of intact leptin–LEPRb signaling in pubertal onset, gonadal function, and reproductive hormone regulation is underscored by the consistency of clinical presentations in affected individuals and by strong evidence from experimental models. Although hormone replacement therapy and assisted reproductive technology continue to be the mainstays of treatment, newer strategies that focus on intervention further downstream in hypothalamic pathways, such as the discovery of kisspeptin, have encouraging prospects for mechanism-based therapies.

Beyond its significance as a rare genetic disorder, LEPRb deficiency provides a valuable framework for understanding reproductive dysfunction associated with acquired leptin resistance in common metabolic conditions such as obesity. To reestablish metabolic–reproductive integration, targeted therapies will require additional developments in the biology of leptin receptors and the manipulation of neuroendocrine circuits.

## Figures and Tables

**Table 1:** Structural domains of the LEPRb and their associated signaling functions. The table summarizes the principal extracellular and intracellular components of LEPRb, their molecular roles in leptin-mediated signal transduction, and their relevance to hypothalamic regulation of reproductive function.

LEPRb Component	Structural Features	Molecular Function	Reproductive Relevance
Extracellular domain [30]	Leptin-binding region; FNIII domains	Ligand recognition and receptor dimerization	Required for leptin-dependent hypothalamic signaling
Transmembrane domain [31,32]	Single-pass membrane segment	Anchors receptor to cell membrane	Enables signal transmission to intracellular domain
Intracellular Box 1 motif [22]	Conserved proline-rich sequence	JAK2 recruitment and activation	Essential for downstream STAT3 signaling
Intracellular Box 2 motif [5,30]	Regulatory signaling region	Stabilizes JAK2 interaction	Supports sustained receptor activation
STAT3 phosphorylation sites [31]	Tyrosine residues (e.g., Tyr1138)	STAT3 activation and nuclear translocation	Regulates Kiss1 and neuroendocrine gene expression
PI3K/Akt interaction sites [33]	Intracellular docking regions	Modulates neuronal excitability	Influences GnRH pulsatility
MAPK/ERK signaling interface [34]	Adaptor protein recruitment	Regulates transcription and neuronal plasticity	Supports long-term neuroendocrine regulation

**Table 2:** Functional classes of LEPRb mutations and their molecular and reproductive consequences. The table summarizes major categories of pathogenic LEPRb variants, the receptor domains affected, resulting signaling defects, and associated reproductive outcomes.

Mutation Type	Affected Domain	Molecular Defect	Signaling Consequence	Reproductive Outcome
Missense [10,45]	Extracellular (FNIII)	Reduced leptin binding	Impaired receptor activation	HH
Missense [46]	Box 1/Box 2	Defective JAK2 recruitment	Reduced STAT3 activation	Delayed/absent puberty
Nonsense [46]	Intracellular domain	Truncated receptor	Loss of signaling	Severe reproductive failure
Frameshift [44,47]	Any domain	Protein instability	Absent or minimal signaling	Early-onset hypogonadism
Splice-site [8]	Various	Aberrant receptor assembly	Reduced surface expression	Variable phenotype
Large deletions [48]	LEPRb	Loss of long isoform	Complete signaling deficiency	Profound infertility

**Table 3:** Summary of representative LEPR mutation categories, affected domains, predicted effects on LEPRb signaling, circulating leptin levels, and pubertal development. Severity of pubertal impairment generally correlates with the degree of disruption of LEPRb-mediated intracellular signaling.

Mutation Category	Affected Domain	Predicted Signaling Defect	Circulating Leptin Levels	Pubertal Development
Biallelic nonsense mutations [44,45]	Intracellular domain (truncating)	Complete loss of JAK2/STAT3 signaling	Markedly elevated	Absent spontaneous puberty
Frameshift mutations [44,46]	Variable (often intracellular)	Truncated or unstable receptor protein; minimal signaling	Elevated	Absent or severely delayed puberty
Missense mutations (FNIII domain) [43,44]	Extracellular leptin-binding domain	Reduced leptin binding affinity	Elevated	Delayed puberty (variable severity)
Missense mutations (Box 1/Box 2 motifs) [10,44]	JAK2 recruitment domain	Impaired STAT3 activation	Elevated	Absent or incomplete pubertal progression
Splice-site mutations [44,46]	Variable	Aberrant receptor assembly; reduced surface expression	Elevated	Variable; may show partial development
Large deletions affecting LEPRb [44,47]	Long signaling isoform	Complete signaling deficiency	Markedly elevated	No spontaneous puberty
Compound heterozygous mutations [39,44]	Mixed domains	Partial residual signaling depending on allele	Elevated	Delayed puberty; heterogeneous

## Data Availability

No new data were created or analyzed in this study.
